# Immunometabolism of macrophages regulates skeletal muscle regeneration

**DOI:** 10.3389/fcell.2022.948819

**Published:** 2022-09-06

**Authors:** Yu-Fan Chen, Chien-Wei Lee, Hao-Hsiang Wu, Wei-Ting Lin, Oscar K. Lee

**Affiliations:** ^1^ Center for Translational Genomics Research, China Medical University Hospital, Taichung, Taiwan; ^2^ Institute of Clinical Medicine, National Yang Ming Chiao Tung University, Taipei, Taiwan; ^3^ Stem Cell Research Center, National Yang Ming Chiao Tung University, Taipei, Taiwan; ^4^ Doctoral Degree Program of Translational Medicine, National Yang Ming Chiao Tung University and Academia Sinica, Taipei, Taiwan; ^5^ Department of Orthopedics, China Medical University Hospital, Taichung, Taiwan

**Keywords:** muscle stem cells, macrophages, muscle regeneration, sarcopenia, metabolism

## Abstract

Sarcopenia is an age-related progressive loss of skeletal muscle mass, quality, and strength disease. In addition, sarcopenia is tightly correlated with age-associated pathologies, such as sarcopenic obesity and osteoporosis. Further understanding of disease mechanisms and the therapeutic strategies in muscle regeneration requires a deeper knowledge of the interaction of skeletal muscle and other cells in the muscle tissue. Skeletal muscle regeneration is a complex process that requires a series of highly coordinated events involving communication between muscle stem cells and niche cells, such as muscle fibro/adipogenic progenitors and macrophages. Macrophages play a critical role in tissue regeneration and the maintenance of muscle homeostasis by producing growth factors and cytokines that regulate muscle stem cells and myofibroblast activation. Furthermore, the aging-related immune dysregulation associated with the release of trophic factors and the polarization in macrophages transiently affect the inflammatory phase and impair muscle regeneration. In this review, we focus on the role and regulation of macrophages in skeletal muscle regeneration and homeostasis. The aim of this review is to highlight the important roles of macrophages as a therapeutic target in age-related sarcopenia and the increasing understanding of how macrophages are regulated will help to advance skeletal muscle regeneration.

## 1 Introduction

### 1.1 Role of macrophages in skeletal muscle

Macrophages originate from monocytes and provide the first line of defense against pathogens. They also play a significant role in initiating inflammation and resolving inflammatory responses (Bosurgi et al., 2011; Chazaud, 2020), which connect the innate and adaptive immunity. During muscle regeneration, macrophages can phagocyte cellular debris, release numerous cytokines and growth factors, and recruit other immune cells. In 1996, different subpopulations of macrophages with distinct spatial and temporal distributions were first observed during skeletal muscle regeneration (McLennan, 1996). Using lineage tracing in sequential stages of skeletal muscle regeneration, lymphocyte antigen 6 complex (Ly6C+) macrophages in the inflammatory stage were shown to switch to Ly6C- macrophages in the regenerative stage (Varga et al., 2013). In addition, Mosser and Edwards proposed the plasticity of macrophages with pro-inflammatory (M1) and anti-inflammatory (M2) polarization responses to cytokine stimulation *in vitro* (Mosser and Edwards, 2008; Liu et al., 2014). However, the underlying molecular regulation of which subset of macrophages regulates skeletal muscle regeneration is still unknown. In the last decade, the role of macrophage dynamics during skeletal muscle regeneration has been widely explored (Saclier et al., 2013; De Santa et al., 2019; Ziemkiewicz et al., 2021). Inflammatory macrophages were identified after muscle damage and produced nitric oxide (NO) by metabolizing l-arginine. Pro-inflammatory macrophages remove cell debris from necrotic muscles during the inflammatory stage (Juban and Chazaud, 2021). For instance, Chakarov et al.(2019) identified two interstitial subsets of macrophages: one population was localized near nerve fibers and supported their differentiation, whereas the other population was preferentially localized near the blood vessels and may support vessel integrity and inhibit inflammatory cell infiltration into tissues. Further, the proliferation of myogenic precursor cells (MPCs) increases upon co-culture with pro-inflammatory macrophages (Arnold et al., 2007). Numerous studies have indicated that anti-inflammatory macrophages promote the differentiation and fusion of MPCs at the restorative stage. Therefore, macrophage phenotypic switching and crosstalk with MPCs are required for skeletal muscle regeneration. These results highlight the functional diversity of macrophages *in vivo*, which may be attributed to their niche. Thus, the further understanding of disease mechanisms of sarcopenia and its therapeutic strategies requires a more profound knowledge of macrophages in skeletal muscle. Muscular diseases are often associated with chronic inflammation and dysregulation of inflammation (Dort et al., 2019). When skeletal muscle regeneration is dysregulated during chronic injury, persistent inflammation mediated by macrophages can result in muscle fibrosis and impair MPCs. Skeletal muscle regeneration is perturbed by asynchronous muscle injuries, leading to the concurrence of pro- and anti-inflammatory macrophages and increased muscle fibrosis (Dadgar et al., 2014). Muscular dystrophy is characterized by progressive weakness and loss of muscle mass associated with genetic deficiency. Dysferlin (Dysf) is a dystrophy-associated fer-1-like protein that participates in skeletal muscle repair. Dysferlinopathy in Dysf-deficient mice skews intramuscular macrophages towards the pro-inflammatory phenotype (Baek et al., 2017). Long-term maintenance of the pro-inflammatory state can cause apoptosis and necrosis of MPCs. Duchenne muscular dystrophy (DMD) is a severe muscle degenerative disease caused by a dystrophin mutation that results in ambulation loss, respiratory dysfunction, and premature death. Dystrophin plays an important role in maintaining the hemostasis of MPCs by controlling polarity and asymmetric division (Dumont et al., 2015b). Clinical data from muscle biopsies of patients with DMD showed highly activated inflammatory signaling, through the nuclear factor kappa B (NF-κB) and transforming growth factor β (TGF-β) pathways (Chen et al., 2005). Mdx mice with a dystrophin deficiency are widely used animal models for studying DMD pathology (Petrof, 2002; Hammers et al., 2020; Saclier et al., 2021), where the loss of NO synthase is observed in dystrophin-deficient muscles (Tidball and Wehling-Henricks, 2014).

### 1.2 Macrophage metabolism and muscle regeneration

The amount of lactate produced by skeletal muscle cells and macrophages after exercise, causes hyperlactatemia thereby implying metabolic remodeling during muscle inflammation (Alamdari et al., 2008; Tékus et al., 2012; Pillon et al., 2013). Pro-inflammatory macrophages, induced by lipopolysaccharide (LPS) and interferon (IFN)γ, display enhanced glycolysis to convert pyruvate to lactate (Van den Bossche et al., 2015; Eshghjoo et al., 2021; Liu et al., 2021). Lactate-polarized macrophages exhibit an anti-inflammatory phenotype and promote muscle revascularization and regeneration (Zhang et al., 2020). Metabolic and transcriptional screening of macrophages with M1 polarization *in vitro* indicated an upregulation of glycolytic genes and downregulation of oxidative phosphorylation (OxPhos)-associated genes. Hypoxia-inducible factor 1-alpha (HIF-1α) signaling is important for regulating glycolysis and is upregulated in M1 macrophages (Jha et al., 2015; Jiang et al., 2017). Enhanced glycolysis and hyper-inflammation with HIF-1α overexpression in macrophages was also characterized by a low oxygen consumption/extracellular acidification ratio and upregulation of pro-inflammatory cytokines, such as inducible nitric oxide synthase (iNOS), interleukin (IL)-6, tumor necrosis factor (TNF)-α, and IL-1β (Wang et al., 2017). The overexpression of glucose transporter 1, the primary time-limiting glucose transporter, led to increased glycolytic activity *via* glucose uptake and secretion of inflammatory cytokines in LPS-induced M1 macrophages (Freemerman et al., 2014). LPS increases the level of succinate, an intermediate of the Krebs/tricarboxylic acid (TCA) cycle in M1 macrophages, because NO production inhibits succinate dehydrogenase (SDH)-mediated conversion of succinate to fumarate (De Santa et al., 2019). Succinate is reported to be an inflammatory signal that increases IL-1β production *via* HIF-1α signaling (Tannahill et al., 2013). In contrast, anti-inflammatory macrophages increase OxPhos, converting pyruvate to acetyl-CoA. Arginase 1 (ARG1) is highly expressed in M2 macrophages and competes with iNOS for the common substrate l-arginine to produce ornithine and urea (De Santa et al., 2019). Since carbohydrate kinase-like (CARKL) could phosphorylate the pentose phosphate pathway intermediate sedoheptulose to sedoheptulose-7-phosphate and is highly upregulated in M2 polarized macrophages, repression of PPP is also considered as a characterization of metabolic features in anti-inflammatory macrophages (Haschemi et al., 2012; De Santa et al., 2019).

**FIGURE 1:**
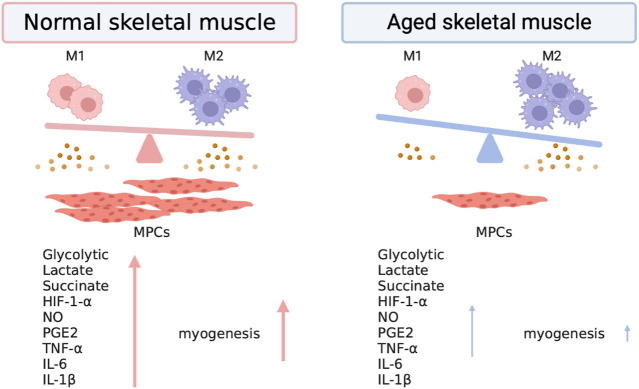
Skewed metabolic regulation and cytokine production in macrophages impairs skeletal muscle regeneration. Pro-inflammatory macrophages exhibit increased glucose uptake and glycolysis, leading to the accumulation of lactate and entry of glucose-derived pyruvate into the TCA cycle. Succinate accumulation in pro-inflammatory macrophages stabilizes HIF-1α, whereas anti-inflammatory macrophages increase the uptake of lipids and augment fatty acid oxidation. Pro-inflammatory macrophages are characterized by glycolytic metabolism, inducible nitric oxide synthase (iNOS) expression, and the production of pro-inflammatory cytokines such as TNF-α, IL-6, and IL-1α. Pro-inflammatory macrophages are critical in promoting myogenic precursor cells (MPCs) to enhance skeletal muscle regeneration. However, anti-inflammatory macrophages are the dominating type of macrophage in aged skeletal muscle; pro-inflammatory macrophages showed lower abundance in skeletal muscle and declined with age (Cui et al., 2019). Therefore, phenotypic and metabolic skewing may impair skeletal muscle regeneration in the elderly population.

Differences in the metabolic profiles of pro- and anti-inflammatory macrophages also cause the accumulation of intermediates (De Santa et al., 2019). Citrate carriers are upregulated and SDH is inhibited by NO in pro-inflammatory macrophages. The flux of the TCA cycle is discontinued in two stages, citrate and succinate, leading to citrate and succinate accumulation in the cytosol. Anti-inflammatory macrophages have intact TCA cycle flux and take up more glucose in the cytosol than unpolarized macrophages, but less than that by pro-inflammatory macrophages. Enhanced fatty acid uptake and fatty acid oxidation (FAO) have been reported in anti-inflammatory macrophages. Pharmacological inhibition of FAO by etomoxir (a mitochondrial CPT1 inhibitor) dramatically reduces ARG1 expression and activity in IL-4-induced M2 macrophages (Huang et al., 2014). Signal transducer and activator of transcription STAT6 and peroxisome proliferator-activated receptor-gamma coactivator (PGC)-1β can activate the FAO pathway to induce the M2 anti-inflammatory phenotype in macrophages (Vats et al., 2006). However, recent studies have indicated that FAO is not essential for the growth of anti-inflammatory macrophages. Nomura et al. demonstrated that etomoxir treatment inhibited ARG1 expression and M2 polarization in FAO-inhibited Cpt2-deleted macrophages (Divakaruni et al., 2018). In addition, transient expression of granulocyte-macrophage colony-stimulating factor in the skeletal muscle after injury increases pro-inflammatory macrophages and promotes myogenesis (Martins et al., 2020). TNF-α is also a pro-inflammatory cytokine and has a mitogenic effect that promotes muscle stem cell (MuSC) and MPCs proliferation (Li, 2003). In TNF receptor 1 and 2 double knockout mice, muscle strength exhibited a deficit, and the expression of myoblast determination protein 1 (MyoD) was reduced after freeze injury (Warren et al., 2002). IL-6, which is mostly derived from macrophages, is important for the regulation of skeletal muscle regeneration via STAT3 (Smith, 2018). IL-6-dependent activation of STAT3 is specifically required for MuSC proliferation *in vitro*. In IL-6 deficient mice, the proliferation of MuSCs was abrogated (Serrano et al., 2008; Zhang et al., 2013), and IL-6 is also necessary for the complete differentiation of muscle cells. IL-1β is mainly secreted by pro-inflammatory macrophages. IL-1β treatment induces the upregulation of MyoD and myogenin in C2C12 myoblast cells (Li et al., 2009). MPCs isolated from IL-1β knockout mice exhibited slower proliferation than those isolated from wild-type mice. However, long-term exposure to IL-1β can result in the reduction of myotubes and loss of sarcomeric actin (Chaweewannakorn et al., 2018). Overall, at the inflammatory stage of skeletal muscle regeneration, pro-inflammatory macrophage-mediated responses are crucial for regulating MuSC activation and MPCs proliferation. IL-10 is an anti-inflammatory cytokine upregulated during the restorative stage of skeletal muscle regeneration. IL-10 treatment did not affect proliferation and MyoD expression in myoblasts. Additionally, IL-10 ablation could cause a delay in pro-to anti-macrophage transition and reduce muscle fiber repair (Villalta et al., 2011; Deng et al., 2012). In the skeletal muscle, reduced glucose uptake may further impair glucose homeostasis and protein synthesis, leading to sarcopenia (Summary in Figure 1). Furthermore, ischemia and aged-related muscle wasting are conditions characterized by reduced glutamine. In a recent study, Shang et al. (2020) demonstrated restrictions of glutamine in the muscle in an aging murine model. Skeletal muscle with low levels of glutamine caused infiltrating macrophages to secrete more glutamine, further promoting MuSC activation in response to injury and sarcopenia during aging. Collectively, these findings highlight the role of glycolytic and oxidative metabolism in the regulation of macrophage function in tissue regeneration. This evidence indicates the importance of exploring potential therapeutic strategies for muscular diseases by investigating metabolic products and intermediates in the metabolic remodeling of macrophage polarization.

### 1.3 Sarcopenia

Sarcopenia was first described by Rosenberg as an important public health problem, causing physical frailty, mobility limitation, and premature death (Rosenberg, 1997). Risk factors for sarcopenia include age, gender, and level of physical activity. There are two core components for diagnosing sarcopenia; the first is the loss of muscle mass, and the second is loss of muscle strength. Notably, frailty is defined by the presence of one or more of the following components: unintentional weight loss, exhaustion, weakness, and low physical activity level (Kim et al., 2021). According to cut-offs from the European Working Group on Sarcopenia in Older People (EWGSOP) and the Asian Working Group for Sarcopenia (AWGS), its prevalence is estimated to be 10–30% worldwide (Chen et al., 2016; Shafiee et al., 2017). Typical features of sarcopenic skeletal muscle are fewer and thinner myofibers as well as altered myofiber type composition toward the type I phenotype (Purves-Smith et al., 2014). The loss of muscle strength is usually evaluated by grip strength, gait speed, and chair stand tests, which remain the simplest and cheapest methods; however, muscle mass can be estimated by radiological techniques, including dual X-ray absorptiometry, computed tomography (CT), or magnetic resonance imaging (MRI), to identify patients at risk for sarcopenia-related morbidities and suggest preventive interventions. Sarcopenic skeletal muscle is highly associated with 1) Loss of the MuSC pool and reduction of regenerative ability, 2) Increases in extracellular matrix (ECM) deposition, 3) Intramuscular adipose tissue infiltration, and 4) Chronic local inflammation (Addison et al., 2014; Chen et al., 2021). In the following sections, we discuss the roles of macrophages in these scenarios as well as their function in skeletal muscle and sarcopenia.

#### 1.3.1 Loss of the muscle stem cells pool and reduction of regenerative ability

Skeletal muscle shows remarkable regenerative ability, including gross structure and functionality, which depends on a unique and rare cell population called MuSCs or satellite cells (Charge and Rudnicki, 2004; Collins et al., 2005; Kuang and Rudnicki, 2008). MuSCs, first described in 1961, are located between the basal lamina and sarcolemma, close to the capillaries, and form at higher densities near neuromuscular and tendon junctions (Mauro, 1961; Relaix, 2006; Bentzinger et al., 2012; Nederveen et al., 2021). MuSCs play indispensable roles in muscle growth and regeneration (Mauro, 1961; Dumont et al., 2015a). Adult MuSCs typically exist in a quiescent state. In steady state conditions or micro-injury conditions (e.g., exercise), MuSC homeostasis is achieved by asymmetric cell division, that is, generating one naïve MuSC to replenish the stem cell pool, and another activated MuSC proliferates to support skeletal muscle regeneration. Upon acute or chronic damage, robust quiescent MuSCs enter the cell cycle and become activated MuSCs. Activated MuSCs have an increased migratory capacity, which enables them to migrate to injury sites, proliferate and differentiate into myoblasts, and then fuse to form new multinuclear myofibers. After complete regeneration, some of the activated MuSCs return to a quiescent state to reconstitute the stem cell pool (Pownall et al., 2002; Rocheteau et al., 2012; Evano and Tajbakhsh, 2018). Interestingly, MuSCs possess both asymmetric and symmetric divisions during muscle regeneration, which is critical for maintaining the stem cell pool and muscle homeostasis for the next challenge (Cossu and Tajbakhsh, 2007; Kuang et al., 2007; Motohashi and Asakura, 2014); however, the mechanisms by which MuSCs fine-tune the balance between asymmetric and symmetric divisions are still under investigation.

The quiescence, activation, proliferation, and differentiation of MuSCs are tightly regulated by sequential changes in skeletal muscle-specific transcription factors, including paired box proteins 3 (Pax3), Pax7, and myogenic regulatory factors (MRFs). MRFs are a family of basic helix-loop-helix transcription factors consisting of myogenic factor 5 (Myf5), MyoD, myogenin, and Myf6 (von Maltzahn et al., 2013; Berberoglu et al., 2017; Ishido et al., 2009; Sambasivan et al., 2011; Zammit, 2017; Asfour et al., 2018). Pax7 is highly expressed in quiescent MuSCs (Pax7+/CD34+) and is required for their survival, self-renewal, and differentiation (von Maltzahn et al., 2013). Upon activation, Myf5 and MyoD levels increase rapidly in MuSCs (Pax7+/Myf5+/MyoD+) initiating proliferation (Cooper et al., 1999; Almada and Wagers, 2016). Activated MuSCs further commit to myoblasts by increasing myogenin and Myf6 and decreasing Pax7, which eventually forces myoblasts to exit the cell cycle and fuse with each other to form new muscle myofibers or fuse with the nearby myofibers (Abmayr and Pavlath, 2012; Zammit, 2017). Reviving the number and function of MuSCs has been considered as a powerful therapeutic approach for muscular dystrophies through muscle regeneration (Sass et al., 2018; Judson and Rossi, 2020; Shcherbina et al., 2020). However, the contribution of declining MuSC pool and their reduced functionality in the initiation and progression of sarcopenia remains controversial. The loss of MuSC number during aging has been shown to lead to the loss of nuclei in large fibers, and reducing the nuclei length might induce cytoplasmic atrophy and sarcopenia (Brack et al., 2005). Loss of adult MuSCs during aging drives aging-related neuromuscular junction degeneration, which induces skeletal muscle loss (Liu et al., 2017). MuSCs are also important for lifelong exercise- (Englund et al., 2020) or overload-induced muscle hypertrophy (Egner et al., 2016; Goh and Millay, 2017; Murach et al., 2017), suggesting that MuSCs are critical for skeletal muscle maintenance and homeostasis. MuSCs contribute to skeletal muscle homeostasis in adults. Although ablation of MuSCs in adult mice impaired skeletal muscle regenerative capacity, it did not cause a reduction in the cross-sectional area of uninjured adult muscle regardless of myofiber types (Fry et al., 2015; Keefe et al., 2015). However, it can be speculated that experimental ablation of MuSCs in mice reduces the bystander effect (da Silva et al., 2019) of aged MuSCs, thereby compromising the effect of their decline on sarcopenia.

Unlike stem cells that undergo rapid turnover, such as hematopoietic stem cells and intestinal stem cells, the quiescent and persistent nature of MuSCs exposes them to genotoxic stresses (Fulle et al., 2005) throughout life. This leads to cellular apoptosis and senescence, a state of irreversible cell cycle arrest, and ultimately causes aging-related decline in the regenerative ability of the skeletal muscle. The functional decline of MuSCs is determined by both intrinsic features, such as heterogeneity, epigenetic signature, and cell signaling, as well as extrinsic features, such as matrix remodeling, mechanotransduction, and communication between different cells within the skeletal muscle (Blau et al., 2015; Hong et al., 2021; Relaix et al., 2021; Sousa-Victor et al., 2021). The effect of immune cells such as macrophages which are the most sensitive population to biological and chronological aging, on muscle regeneration has not been addressed extensively.

#### 1.3.2 Increases in extracellular matrix deposition

Efficient muscle repair requires the proliferation of fibroblasts to produce ECM components, such as collagen, fibronectin, elastin, proteoglycans, and laminin (Frantz et al., 2010). The former components serve to stabilize the muscle tissue and act as a scaffold for the new muscle fibers. However, increased collagen and ECM deposition will cause fibrosis, scar formation, and impair skeletal muscle function. Moreover, abnormalities in ECM production and remodeling contribute to tissue dysfunction. Fibrosis affects a wide range of tissues and includes cellular and molecular mechanisms such as degeneration, infiltration of leukocytes, persistent inflammation, and proliferation of cells that resemble fibroblasts. Cell-signaling pathways are also under a perpetual remodeling process influencing ECM formation. Transforming growth factor β (TGF-β) activates a signaling pathway for cell proliferation, differentiation, and development and can promote collagen synthesis for wound healing. The Notch signaling pathway has also been strongly implicated in aging-associated fibrosis. For example, Carlson et al. (2008) demonstrated that TGF-β is increased in the niche of aged murine MuSCs with reciprocal levels to active Notch, which is more abundant in the young niche. Moreover, Mourikis et al. (2012) demonstrated that activation of Notch signaling can promote MuSC self-renewal and proliferation and inhibits their differentiation into the myogenic lineage through repressing MyoD. In addition, Wnt3A stimulation negatively modulated cell proliferation in young regenerating muscles and augmented fibrosis. Thus, these results imply that aging was associated with alterations in the systemic environment and, because the effects were reversible, provide the strategic basis for interventions aimed at improving tissue repair and at reducing fibrosis in pathological conditions. Interestingly, a recent study demonstrated that age-associated changes in the ECM might be regulated by metalloproteinase 2 (MMP-2) activity. Furthermore, Peck et al. (2022) identified macrophages as a source of MMP-14 in skeletal muscle, which can promote ECM remodeling in response to mechanical loading.

#### 1.3.3 Intramuscular adipose tissue infiltration

In skeletal muscle, aging is accompanied by intramuscular fat infiltration. In addition, muscle fat infiltration is a common feature in several myopathies and is associated with muscular dysfunction and insulin resistance (Fujiwara et al., 2015). Studies have shown that excessive intermuscular fat accumulation decreases muscle strength and interferes with insulin sensitivity and lipid metabolism; thus, intramuscular fat may contribute to muscle weakness (Ahmed et al., 2018). There could be a feedback effect between the whole-body metabolism and local intramuscular fat as a characteristic of many diseases. In addition, intramuscular fat infiltration may be significantly correlated with other diseases, such as cardiovascular disease (Huynh et al., 2022). Considering the prevalence of intramuscular fat and its association with muscular dysfunction and related diseases, it is critical to understand the regulatory mechanisms governing intramuscular adipose tissue infiltration and their impact on lipid metabolism in skeletal muscle; this knowledge may aid in the development of innovative treatments for combating these pathological conditions. However, the cellular origin and lipidomic and transcriptomic changes during fat infiltration in skeletal muscle remain unclear. Intramuscular adipose tissue and sarcopenia may adversely impact mobility function and physical activity. Macrophages are crucial mediators of chronic inflammation, infiltrating obese adipose tissue and stimulating metabolic disorders (Li et al., 2022). Adipose tissue macrophages (ATMs) are thought to be formed from accumulated circulating monocytes in the adipose tissue, self-renewal of tissue-resident macrophages, or *in situ* proliferation under the influence of monocyte chemotactic protein 1 (MCP-1). Recent studies demonstrated that intramuscular adipocytes mainly emanated from a population of fibro/adipogenic progenitors (FAPs) that reside between muscle fibers (Joe et al., 2010). Their role in muscle regeneration was, in part, elucidated in mice. After injury, FAPs proliferate, interact with myoblasts to promote the formation of new muscle fibers, and eventually return to a quiescent state or are cleared by apoptosis. However, the mechanisms controlling their adipogenic potential are still elusive. In a recent study, Moratal et al. (2018) revealed that IL-1β+ (M1-prone) macrophages released cytokines that inhibit FAP adipogenesis via Smad2 phosphorylation, whereas IL-4+ (M2-prone) macrophages had a pro-adipogenic effect.

#### 1.3.4 Chronic local inflammation

Sarcopenia is caused by the catabolism of muscle proteins due to inflammation. Metabolism and immunity are two fundamental systems of metazoans and there appears to be ongoing crosstalk between these two regulatory systems as immune cells, such as macrophages, are present in metabolic tissues. Inflammatory mediators affect muscle protein metabolism, but their exact effects and signaling pathways are unclear (Frasca and Blomberg, 2016). In a recent study, Sciotati et al. (2020) demonstrated that circulating concentrations of IL-6 and TNF-α were significantly elevated in the sarcopenic elderly, and it was reported that higher IL-6 and C-Reactive Protein (CRP) levels increased the risk of muscle strength loss. It is known that exogenous TNF-α, also known as cachectin, impairs muscle function and promotes tissue loss in skeletal muscle (Lang et al., 2002). Additionally, TNF-α reduces the expression of anabolic hormones and growth factors, reduces MyoD and myogenin expression in regenerating muscles, and increases MyoD degradation in myoblasts. Of note, aged mice with genetic TNF-α ablation exhibited reduced sarcopenia and better satellite cell activation (Wang et al., 2018). Asoudeh et al. also demonstrated that inflammatory biomarkers, such as IL-6, TNF-α, and CRP levels, were not associated with sarcopenia in a human blood sample study. Thus, further studies are required to confirm the inflammatory factors in skeletal muscle profiles.

### 1.4 Metabolic regulation in aging

All cellular and biological reactions are fueled by metabolism, including growth, proliferation, differentiation, and autophagy (DeBerardinis et al., 2008; Rabinowitz and White, 2010). The utilization and storage of nutrients is a tightly regulated process that allows cells to maintain nutritional balance and achieve systemic homeostasis in the human body (Efeyan et al., 2015). For example, excess nutrients are converted and stored in the adipose tissue, liver, and skeletal muscle. Skeletal muscles are not only viewed as organs responsible for locomotion but also as the primary site for glucose uptake/storage (Hargreaves and Spriet, 2020). An injury or muscular disease results in muscle degeneration, where the skeletal muscle initiates an inflammatory response that activates immune cells within that area (Arnold et al., 2007). Thus, a better understanding of the metabolic regulation of immune cells in skeletal muscle might provide new insights for modulating muscle diseases, such as sarcopenia.

The phenomenon of aging, which we broadly define as the progressive loss of function with age, affects most living organisms. In the field of aging, many critical questions have been raised based on the conceptual framework concerning the physiological factors that lead to aging-induced damage, compensatory responses that restore homeostasis, the interlinkages between different types of damage and compensatory responses, and the possible exogenous interventions to delay aging. The somatotropic axis in mammals comprises the growth hormone, produced by the anterior pituitary, and its secondary mediator, insulin-like growth factor 1 (IGF-1), which is produced in response to growth hormone by many cell types, notably the hepatocytes. In model organisms, genetic mutations that reduce the functions of the IGF-1 receptor, insulin receptor, and downstream intracellular effectors, such as Akt, mammalian target of rapamycin (mTOR), or forkhead box (FOX), have been linked to longer lifespans, further illustrating the critical role of trophic and bioenergetic pathways in longevity (Johnson et al., 2013). Furthermore, IGF plays an essential role in the regulation of cell growth, differentiation, metabolism, and function. DiToro et al. demonstrated that IGFs regulate Th17, regulatory T (Treg) cells and type 3 innate lymphoid cells by modulating inflammation (DiToro et al., 2020). In addition to the IGF-1 pathway, which helps detect glucose levels, three other related and interconnected nutrient-sensing pathways are being investigated: mTOR, which senses high amino acid concentrations; AMP-activated protein kinase (AMPK), which detects low energy states by sensing high AMP levels; and sirtuins, which detect low energy states by sensing high NAD levels. Interestingly, mice genetically modified to have low mTORC1 activity with no change in mTORC2 and those with S6 Kinase 1 (S6K1—the primary mTORC1 substrate) deficiency are shown to live longer, suggesting that the downregulation of mTORC1/S6K1 is a mediator of longevity (Arif et al., 2017). Furthermore, direct injection of rapamycin into the hypothalamus reverses age-related obesity by increasing mTOR activity (Yang et al., 2012). Taken together, these findings suggest that intensive trophic activity, indicated by the IGF-1 or mTORC1 pathway, accelerates aging. Further, evidence indicates that AMPK activation may mediate lifespan extension following metformin administration in worms and mice (Kulkarni et al., 2020). Thus, these age-related changes in immune defense, triggering low-grade inflammation and metabolic disorders in different immune cells have been discussed further.

## 2 Conclusion and perspectives

Here, we discuss the different metabolic responses employed by macrophages and their importance in regulating the immune system. Activation of macrophages by environmental signals leads to dramatic reprogramming of cellular metabolism. The primary goal of metabolic reprogramming is to provide immune cells with sufficient energy (ATP) and metabolic intermediates to perform their effector functions in maintaining tissue homeostasis. Furthermore, metabolic reprogramming represents a checkpoint before adopting a new cell fate or exerting effector functions. Therefore, interfering with or enhancing specific metabolic programs may be clinically valuable in suppressing pathogenic autoimmunity or chronic inflammation in various metabolic and degenerative diseases. In normal physiological conditions, the immune system is essential in protecting the body from pathogens and tissue repair. When the body is infected by pathogens, inflammation is triggered by immune cells such as macrophages, dendritic cells, and Th cells. A decline in testosterone, growth hormones, androgens, and estrogen occurs with age, dropping the body’s balance towards chronic inflammation, as evidenced by increased blood levels of proinflammatory mediators, such as TNF-α, IL-6, and CRP. It has been shown that macrophages from different tissues possess diverse transcription profiles, impacting their phenotype and function. Due to their tissue-specific functions, macrophages exhibit different transcriptional profiles associated with heterogeneous phenotypes, making them potential therapeutic targets.

Despite rapid progress over the past decade, our understanding of macrophage metabolism is still in its infancy. In most cases, studies have focused on the most abundant cell populations found in the blood or bone marrow rather than on tissue-resident or recruited cells involved in tissue homeostasis. Additionally, current studies have investigated the diversity of immune cell lineages or their tissue-specific functions. Although macrophages and DCs activated by LPS switch from mitochondrial to glycolytic metabolism, it is unclear whether similar metabolic reprogramming occurs in tissue-resident cells *in vivo*, thereby impinging on phagocytic and processing functions. Therefore, single-cell sequencing, in combination with metabolomic studies, is essential to explore the metabolic basis of immunity across the entire spectrum of innate and adaptive immune responses. In particular, the transcriptional coupling of metabolism to cellular effector responses is not unique to immune cells, as previous studies have identified similar critical roles for metabolic regulators, including HIF-1α, PPARγ, AMPK, and PGC-1α in skeletal muscle. Role of fiber-type conversion and exercise tolerance. These findings suggest that metabolic control of immune responses represents a broader paradigm in biology, where the identity, function, and fate of a cell depend on its underlying metabolic state.

## Glossary

MuSCs: muscle stem cells
PAX3: paired box 3
PAX7: paired box 7
MRFs: myogenic regulatory factors
Myf5: myogenic factor 5
Myf6: myogenic factor 6
MyoD: myoblast determination protein 1
IL-1: interleukin-1
IL-1β: interleukin-1-beta
IL-4: interleukin-4
IL-6: interleukin-6
IL-8: interleukin-8
IL-10: interleukin-10
IL-33: interleukin-33
CCL2: C-C motif chemokine ligand 2
mTOR: mammalian target of rapamycin
ILC3: type 3 innate lymphoid cells
S6K1: ribosomal protein S6 kinase beta-1
NADPH: nicotinamide-adenine dinucleotide phosphate
NOX: NADPH oxidases
PPP: pentose phosphate pathway
ROS: reactive oxygen species
DMD: Duchenne muscular dystrophy
TNF: tumor necrosis factor
STAT3: signal transducer and activator of transcription 3
STAT6: signal transducer and activator of transcription 6
PI3Ks: phosphoinositide 3-kinases
Akt: protein kinase B
Ly6c: lymphocyte antigen 6 complex
NO: nitric oxide
MPCs: myogenic precursor cells
LPS: lipopolysaccharide
IFNγ: interferon-gamma
CARKL: carbohydrate kinase-like
FAO: fatty acid oxidation
PGC-1α: PPARγ-coactivator-1α
PGC-1β: PPARγ-coactivator-1β
UDP-GlcNAc: UDP-N-acetylglucosamine
AMPKα1: AMP-activated protein kinase-α1
ARG1: arginase 1
Dysf: dysferlin
NF-κB: nuclear factor kappa B
TGFβ: transforming growth factor beta
DCs: dendritic cells
PPARγ: peroxisome proliferator-activated receptor gamma
CPT1: carnitine palmitoyltransferase I
IGF-1: insulin-like growth factor
